# Supplementary material for: Neisseria gonorrhoeae scavenges host sialic acid for Siglec-mediated, complement-independent suppression of neutrophil activation
Source: mBio. 2024 Apr 9;15(5):e00119-24. doi: 10.1128/mbio.00119-24 (PMC11078009; doi:10.1128/mbio.00119-24)
Supplement: Supplemental Figures — Figures S1 to S4. [file mbio.00119-24-s0001.pdf]

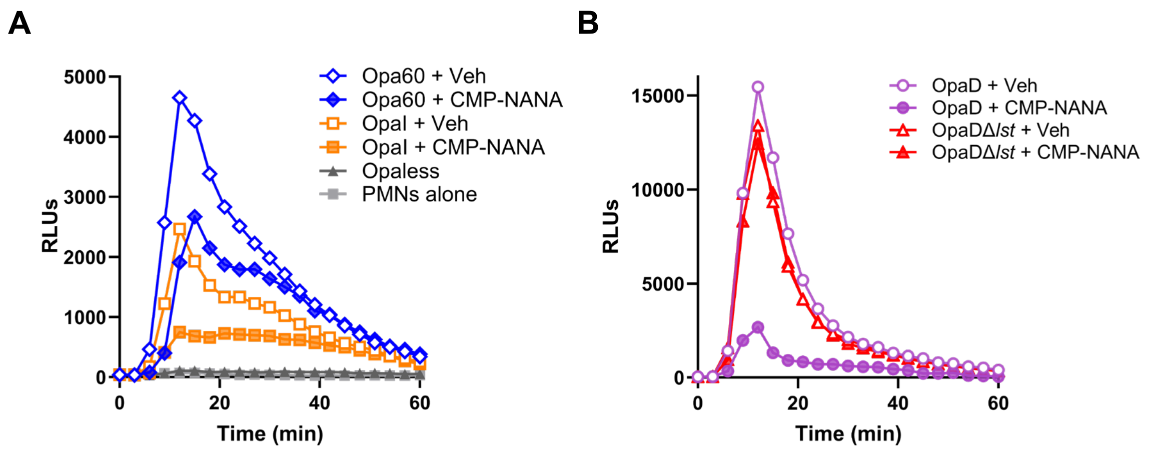


**Fig S1. LOS sialylation reduces neutrophil oxidative burst in response to other Opa+ Gc. (A)** Opa60+ (blue) or OpaI+ (orange) with (filled) or without (empty) sialylation were added to primary human neutrophils at a MOI = 100, in the presence of luminol for chemiluminescence. Reactive oxygen species (ROS) were measured as relative light units (RLUs) every 3 minutes for 1 hour. Uninfected (light grey) and Opaless infected (dark grey) neutrophils (PMNs) serve as negative controls. One replicate of the composite graph in **Fig. 2C** is presented. **(B)** One replicate from the composite graph in **Fig. 2D** is presented, depicting the neutrophil ROS response to OpaD (purple) or OpaD*Δlst* Gc (red) with or without CMP-NANA pre-incubation.

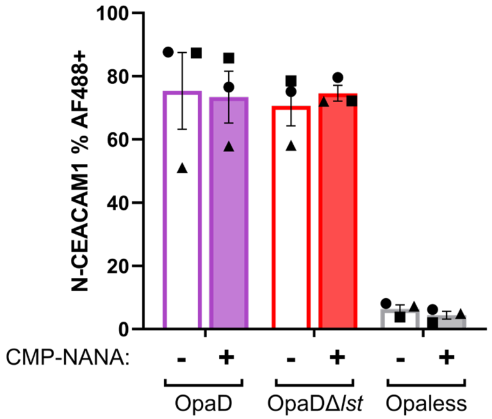


**Fig S2. LOS sialylation does not affect NCEACAM-1 binding to OpaD+ Gc.** OpaD WT (purple), OpaD*Δlst* (red), or Opaless (grey) Gc grown with CMP-NANA (filled) or vehicle (empty) were incubated with GST-tagged recombinant N-terminal domain of CEACAM-1 (NCEACAM-1). Binding of NCEACAM-1 to Gc was measured using a mouse anti-GST antibody, followed by a goat anti-mouse IgG antibody coupled to AF488. Gc were fixed and stained with DAPI before analysis via imaging flow cytometry, and the percent of singlet Gc that are AF488+ was calculated as in **Fig. 4A**. Results are from n=3 biological replicates (symbol matched). Statistical comparisons were by two-way ANOVA with Tukey's multiple comparisons test; not significant.

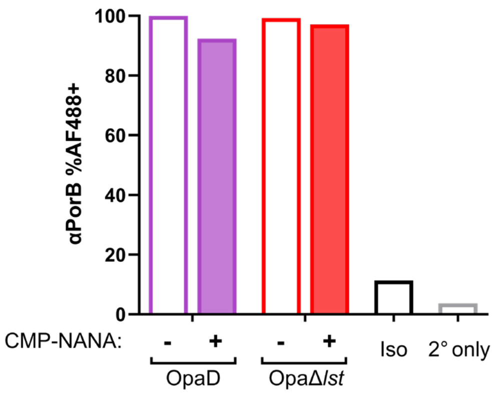


**Fig S3. mAb against FA1090 PorB can be used to detect both sialylated and nonsialylated Gc using imaging flow cytometry**. OpaD WT (purple) or Δ*lst* (red) Gc were treated with (filled) or without CMP-NANA (empty) before staining with TIV, then incubated with murine anti-PorB antibody (purple and red), isotype control (iso; black), followed by goat anti-mouse IgG AF488 secondary antibody. Sialylated OpaD incubated with secondary antibody alone shown in grey (2° only, light grey).

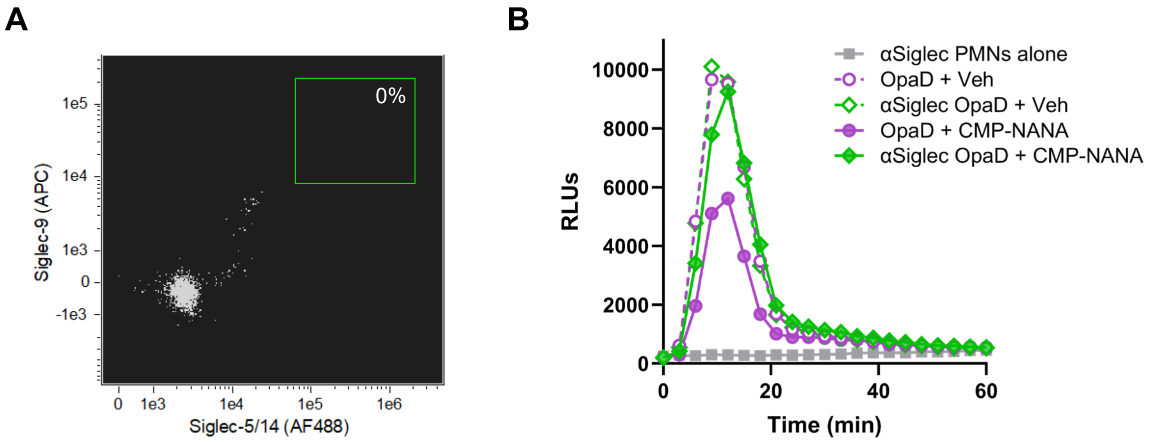


**Fig S4. Blocking Siglec engagement restores the neutrophil oxidative burst in response to Opa+ bacteria.** (**A**) Dot plot of focused singlet neutrophils (uninfected and adherent) that were left unstained but treated as in **Fig 5A**. X-axis indicates AF488 (Siglec-5/14) fluorescence intensity; y-axis indicates APC (Siglec-9) fluorescence intensity. (**B**) Primary human neutrophils were incubated with media alone (purple) or anti-human Siglec-9 + anti-human Siglec-5 and Siglec-14 antibodies (green) for 30 min before being exposed to sialylated (filled) or nonsialylated (empty) OpaD at an MOI of 100 in the presence of luminol. ROS production detected by luminol-dependent chemiluminescence expressed as RLUs over 1 h. Uninfected neutrophils treated with the antibodies serve as negative control (light grey). Graph is a representative of n=6, depicted as composite in **Fig. 5E**.
